# Evaluation of rapid extraction and isothermal amplification techniques for the detection of *Leishmania donovani* DNA from skin lesions of suspected cases at the point of need in Sri Lanka

**DOI:** 10.1186/s13071-018-3238-1

**Published:** 2018-12-22

**Authors:** Gayana Gunaratna, Aresha Manamperi, Susanne Böhlken-Fascher, Renu Wickremasinge, Kithsiri Gunawardena, Bandujith Yapa, Nishantha Pathirana, Hasantha Pathirana, Nilanthi de Silva, Monica Sooriyaarachchi, Theja Deerasinghe, Dinesh Mondal, Shalindra Ranasinghe, Ahmed Abd El Wahed

**Affiliations:** 1 Department of Parasitology, Faculty of Medicine, University of Kelaniya, Ragama, Sri Lanka; 2 Molecular Medicine Unit, Faculty of Medicine, University of Kelaniya, Ragama, Sri Lanka; 3 Microbiology and Animal Hygiene, University of Goettingen, Goettingen, Germany; 4 Department of Parasitology, Faculty of Medical Sciences, University of Sri Jayewardenepura, Gangodawila, Nugegoda, Sri Lanka; 5 Dermatology Unit, District General Hospital Matara, Matara, Sri Lanka; 6 Army Hospital Narahenpita, Narahenpita, Sri Lanka; 7 Dermatology Unit, District General Hospital Hambantota, Hambantota, Sri Lanka; 8 International Center for Diarrheal Disease Research, Dhaka, Bangladesh

**Keywords:** Recombinase polymerases amplification, *Leishmania donovani*, Point of need, Molecular assay

## Abstract

**Background:**

Leishmaniasis is a disease caused by vector-borne protozoans. In Sri Lanka, the cutaneous form of the disease is predominant, which is usually diagnosed using Giemsa-stained slit skin smear examination and by histology. However, the sensitivity of slit skin smears and histology are reportedly low. Moreover, facilities for the highly sensitive polymerase chain reaction (PCR) are available only in a few highly-equipped parasitology laboratories. Therefore, there is a need for low cost, sensitive and specific screening tests for diagnosis of leishmaniasis at the point of need.

**Results:**

In this study, a mobile suitcase laboratory applying novel extraction (SpeedXtract) and isothermal amplification and detection (recombinase polymerase amplification assay, RPA) methods were evaluated for the diagnosis of cutaneous leishmaniasis in Sri Lanka. First, the developed assay was applied to three different sample types (punch biopsy, slit skin smears and fine needle aspirates) at a local hospital. The results showed that the 2 mm punch biopsy sample produced the best exponential amplification curve and early fluorescence signal in the RPA assay. Secondly, punch biopsies were collected from 150 suspected cutaneous leishmaniasis cases and screened with SpeedXtract/RPA, RNA*later*/PCR and ATL buffer/PCR, in addition to Giemsa-stained slit skin smears. Fifty-seven samples were negative in all detection methods. In total 93 samples were positive with assay sensitivities of 65.5% (SpeedXtract/RPA), 63.4% (RNA*later*/PCR) and 92.4% (ATL buffer/PCR). The Giemsa-stained slit skin smear delivered the worst clinical sensitivity (32.2%).

**Conclusions:**

The SpeedXtract/RPA method under field conditions took 35 min, while almost 8 h were needed to finalize the extraction and detection by PCR in the laboratory. The SpeedXtract/RPA method produced similar sensitivity to samples preserved in RNA*later* and subjected to PCR amplification, but both were less sensitive than ATL-preserved samples subjected to PCR amplification. There is a need for a standardization of sample collection and nucleic acid extraction methods.

**Electronic supplementary material:**

The online version of this article (10.1186/s13071-018-3238-1) contains supplementary material, which is available to authorized users.

## Background

Cutaneous leishmaniasis (CL) is a disfiguring neglected tropical disease that affects 1.5 million people annually in the tropics and subtropics [1]. The disease is caused by a protozoan parasite belonging to genus *Leishmania*, and is transmitted by a bite of an infected sand fly. The disease gives rise to a spectrum of clinical presentations ranging from self-limiting lesions to non-healing chronic ulcers. Whatever the range of the clinical spectrum, CL lesions tend to heal with a disfiguring scar [2]. Early accurate diagnosis and treatment will minimize the scar. It is known that CL is poverty-driven, associated with poor housing conditions, living in rural settings with limited resources, animal rearing, etc. [3]. Accurate and rapid diagnosis at the point of care is one of the most difficult challenges encountered in the control of CL. The slit skin smear method is cheap and easy to perform but has shown to be less sensitive with a reported range of sensitivity of 35–70% depending on infective *Leishmania* spp. Histopathology and culture have low detection rates [3, 4]. In addition, histopathogy is time consuming, requires expertise, and most of the culture media are expensive and difficult to prepare. A recently marketed antigen detecting rapid diagnostic test (CL-Detect, In-Bios, Seattle, USA) was shown to have > 90% sensitivity and specificity rates with *Leishmania major* but showed low sensitivity rates (28.4%) in detecting CL due to *Leishmania donovani* (LD) in Sri Lanka [5, 6].

In Sri Lanka, locally acquired CL is solely caused by a naturally attenuated LD Mon-37 strain [7, 8]. The naturally appearing low parasite loads present in CL lesions, PKDL lesions, mucocutaneous lesions or in blood (in VL) make parasitological diagnosis less sensitive by conventional methods [3, 6, 9]. Although no gold standard has yet been identified for the diagnosis of leishmaniases including CL, DNA amplification-based diagnosis has shown the highest sensitivity rates [3]. However, PCR-based diagnostic methods are time consuming, expensive, difficult to perform and require trained staff and high technology equipment. In the scope of overcoming these difficulties while maintaining high sensitivity and specificity, a recombinase polymerase assay (RPA)-based mobile suitcase laboratory was introduced to detect several infectious diseases including visceral leishmaniasis (VL) at the point of care [10]. The mobile laboratory contains a quick DNA extraction (SpeedXtract) and detection method (RPA) that had shown high sensitivity and specificity in detecting *Leishmania* DNA in the blood of patients with VL. However, the efficacy of this method with CL had not yet been evaluated. Therefore, this study aims determination of the most suitable skin specimen for this rapid extraction and RPA-based rapid detection method. The system was then evaluated by screening for 150 suspected CL cases at the point of need in six cities in Sri Lanka.

## Methods

### Sample collection

In order to determine the best sample to perform the SpeedXtract-RPA assay, three different sample types [2 mm punch biopsy, slit skin smear (SSS) and fine needle aspirate (FNA)] were each collected from six suspected CL cases. Thereafter, three 2 mm punch biopsies and one slit skin smear were collected from 150 suspected CL lesions (age, 19–74 years; mean, 41.4 years) between March 2017 and January 2018 to validate the mobile setup at the point of care. All biopsies were collected as close as possible to each other. Only one set of sample collection was done per patient. Of these patients, 27 were from Mulative, 12 from Kilinochchi, 30 from Hambanthota, 8 were referred to Colombo from Northern Province, 12 from Thabuththegama, and 61 from Matara.

### Conduction of SpeedXtract and RPA in a mobile suitcase laboratory

**Fig. 1:**
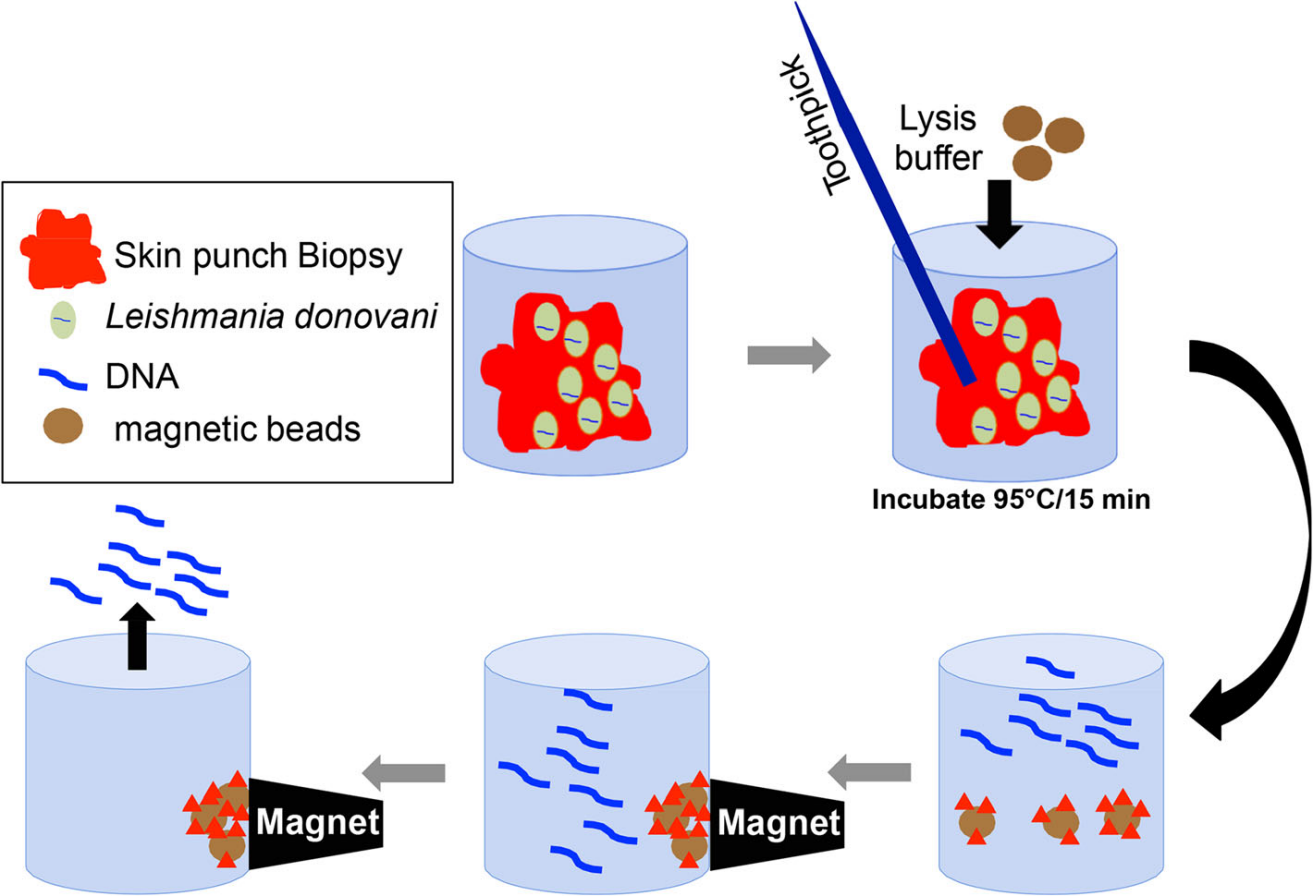
Diagram of the nucleic acid extraction protocol from the skin punch biopsy using SpeedXtract. SpeedXtract is a reverse semi-purification method in which a nucleic acid is extracted by combining lysis buffer and heat. The magnetic beads capture cell membrane, protein and other inhibitors in the sample, while the nucleic acid is free in the supernatant

**Fig. 2:**
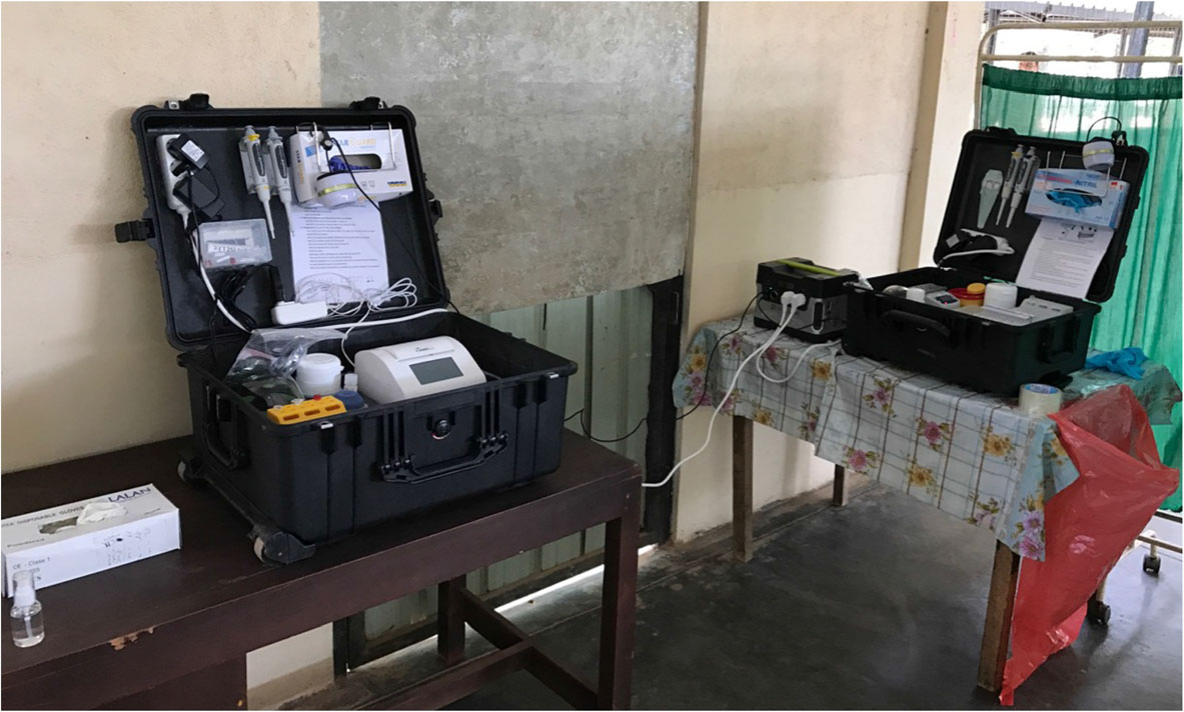
Mobile suitcase laboratory at point of need in Sri Lanka. Right suitcase: DNA extraction using the SpeedXtract kit was performed in the extraction suitcase laboratory in 17 min. Left suitcase: the RPA assay was conducted in 18 min in another suitcase laboratory to avoid cross-contamination

The SpeedXtract (Qiagen, Hilden, Germany) was performed in the extraction suitcase as follows (Figs. 1 and 2): in a low DNA-bind 1.5 ml tube, 100 μl of the lysis buffer was incubated with the collected sample and 30 μl of magnetic beads at 95 °C for 10 min. For the punch biopsy, a toothpick was used to grind the sample after 5 min of incubation. Then the tube was placed in a magnetic stand to separate the magnetic beads. Five microliters of the supernatant was used in the RPA reaction.

The kinetoplast minicircle DNA RPA assay was conducted in the detection suitcase (Fig. 2) as follows: 29.5 μl of the rehydration buffer, 13 μl of the RPA primers (420 nM, FP3: 5'-ATG GGC CAA AAA CCC AAA CTT TTC TGG TCC TC-3' and RP3: 5'-CTC CAC CCG ACC CTA TTT TAC ACC AAC CCC CAG T-3') and probe (120 nM, CGC CTC GGA GCC GAT (BHQ1dT) (Tetrahydrofuran) (FAMdT) TGG CAT TTT TGG CTA TTT TTT GAA CGG GAT-phosphate) [10], 2.5 μl of 14 mM Mg acetate and 5 μl of SpeedXtract supernatant, were added into the lid of the 0.2 ml tube of the RPA lyophilized pellet (TwistAmpexo kits, TwistDx, Cambridge, UK). The tube was closed, centrifuged, vortexed and centrifuged again before it was placed into the portable fluorescence reader (T8 Axxin, Fairfield, Australia) and incubated for 15 min at 42 °C. The fluorescence intensities were measured every 20 s and displayed on the integrated touch screen of the T8 device. An RPA run was considered valid when a clear exponential curve was recorded for the positive control and the negative control showed a clear linear line below 250 mV. Samples were considered positive if they produced an exponential curve above the cut-off value of 300 mV. A 10^5^ linearized molecular DNA standard representing 1– 310 nt of LD kinetoplast minicircle DNA (GenBank accession number: Y11401.1) ordered from GeneArt (Invitrogen, Darmstadt, Germany) was used as the RPA positive control.

### Standard diagnostic methods

At the local hospital, Giemsa-stained slit smears were prepared and examined under light microscopy as described previously [6]. At the parasitology laboratory in University of Sri Jayewardenepura, Sri Lanka, DNA was extracted from collected samples using DNeasy blood and tissue kit (Qiagen, Hilden, Germany) as per the manufacturer’s instructions and conventional PCR was applied as mentioned earlier [11, 12]. In this study we also compared the efficiency of two different types of sample transport media for PCR: ATL buffer that comes with the DNeasy Blood and tissue kit (Qiagen, Hilden, Germany) and RNA*later* (Sigma-Aldrich, Missouri, USA). ITS1-based PCR primers, reagents and conditions were used as previously described [11, 12].

## Results

### Selection of the most suitable skin specimen for rapid extraction and detection by RPA

**Fig. 3:**
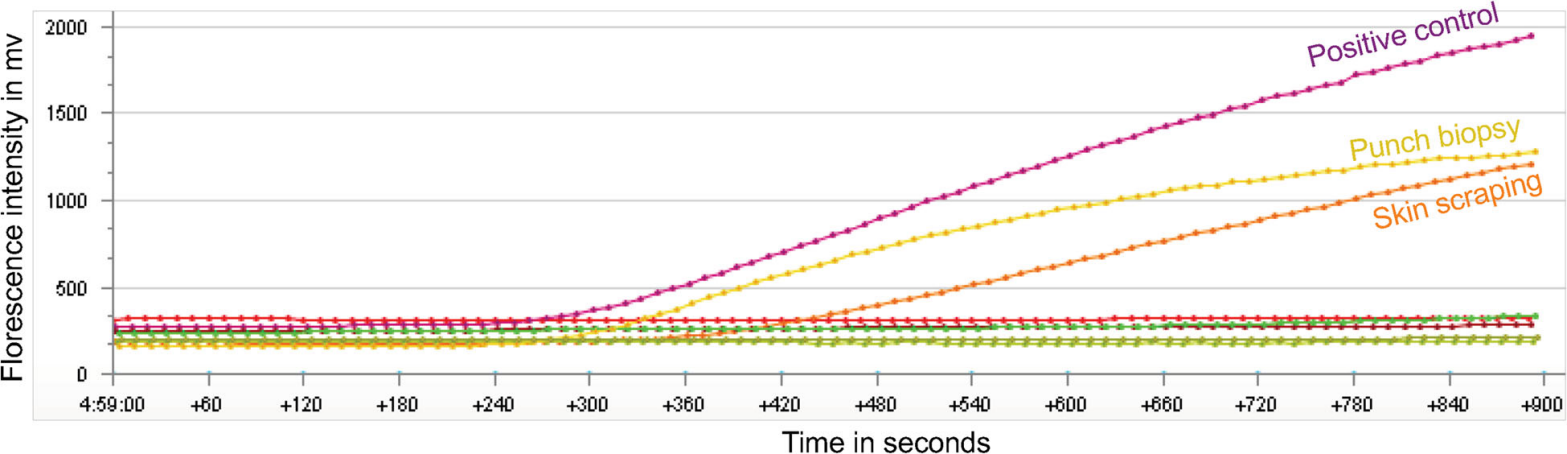
Selection of the best target sample for the RPA assay. Results of screening the skin punch biopsy, scraping and fine needle aspirates from patient numbers 4 and 5 to determine which sample would give the best results with SpeedXtract and RPA at the point of need. The 2 mm punch biopsy sample of patient 5 produced the best exponential amplification curve and early fluorescence signal in RPA assay. All samples from patient 4 were negative. The graph was produced by T8 desktop software (Axxin, Fairfield, Australia)

**Fig. 4:**
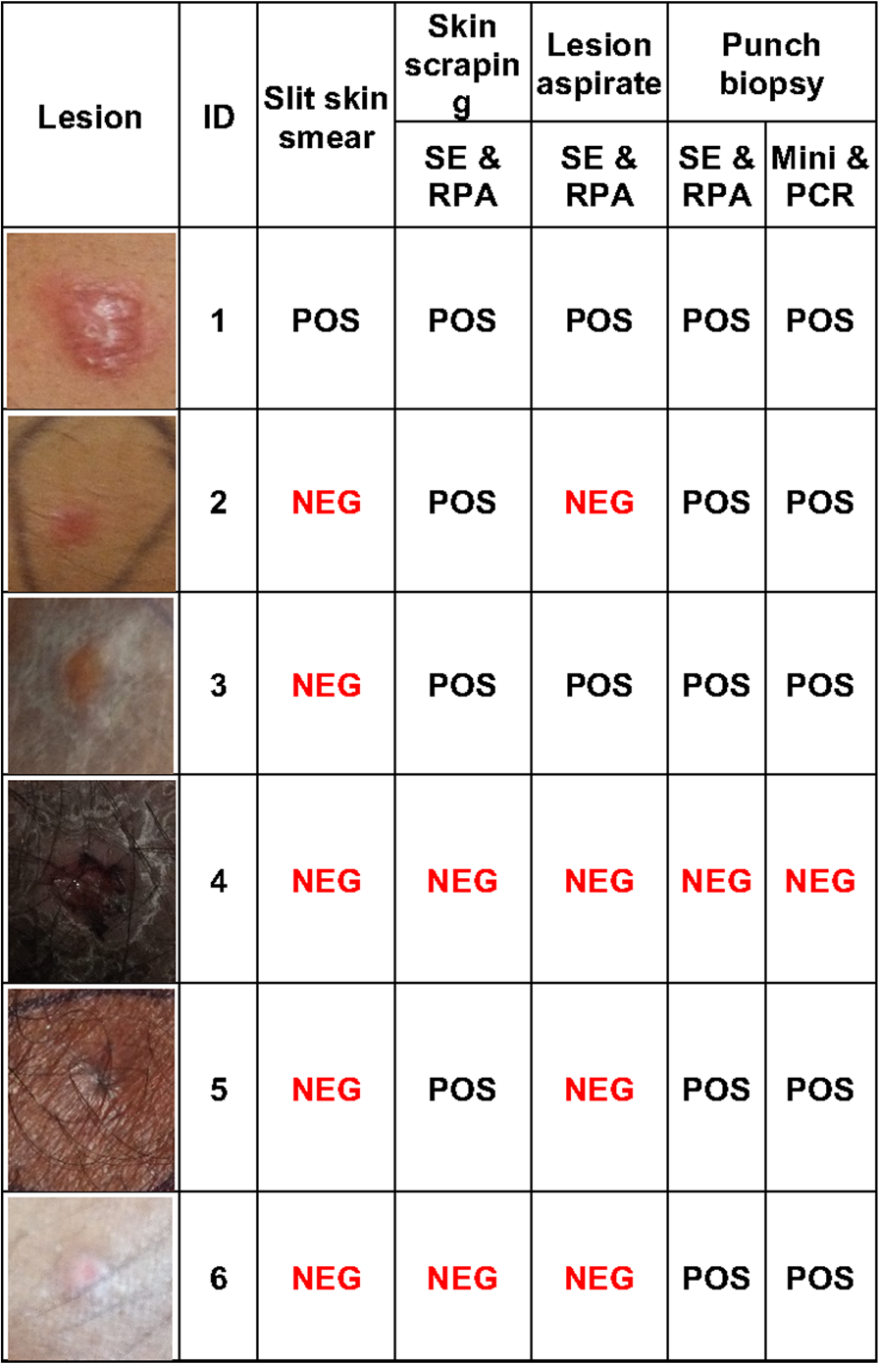
Comparison between the sample types and test methods. The slit skin smears were examined with microscopy. The skin scrapping and aspirate were tested at the point of need with SpeedXtract (SE) and recombinase polymerase amplification (RPA) assay. The punch biopsies were screened with SE and RPA as well as DNeasy blood and tissue kit (mini) and polymerase chain reaction (PCR). *Abbreviations*: ID, sample identification code; POS, *Leishmania donovani* was detected; NEG, negative test results

In field settings, punch biopsy, skin scraping and FNA from six LD suspected cases were screened with SpeedXtract and RPA, in addition to a Giemsa-stained slit skin smear. The punch biopsies were shipped to the central laboratory for DNA extraction using the DNeasy blood and tissue kit and performing PCR. The obtained results are summarized in Figs. 3 and 4. Out of the six suspected CL lesion samples tested, the slit skin smear was positive in only one sample, while five punch biopsies were positive using both RPA and PCR. Remarkably, the test was performed in the field in 35 min, while almost 8 h were required for the laboratory testing, not including the shipment duration of one day. Under field conditions, the punch biopsy produced the best assay sensitivity (5/6), while lower sensitivities were observed by skin scraping (4/6) and lesion aspirate (2/6); these were still notably better than the slit skin smear (Figs. 3 and 4, and Additional file 1: Figure S1).

### Clinical performance of SpeedXtract and RPA at field level

As shown above, the punch biopsy was the sample of choice to get the best assay diagnostic sensitivity; therefore, skin biopsies from 150 suspected CL cases were collected and screened directly on-site using SpeedXtract and RPA, in addition to a Giemsa-stained slit skin smear. Two additional skin biopsies from the same patient were preserved in either RNA*later* or ATL buffer and shipped to the central laboratory for DNA extraction using the DNeasy blood and tissue kit and detection using conventional PCR. The results are included in Additional file 2: Table S1. In total, 57 samples were negative in all detection methods. The Giemsa-stained slit skin smear delivered the worst clinical sensitivity (32.2%) as only 30 samples were positive compared to a total of 93 positive samples confirmed through PCR. Comparatively, RPA and PCR using RNAlater-preserved samples produced sensitivities of 65.5 and 63.4%, respectively. PCR using ATL-preserved samples had the best sensitivity (92.4%). All SSS positives were always positive by ATL buffer/PCR and all RPA were also positive by ATL buffer/PCR (Table 1).

**Table 1:** Analysis of diagnostic assays for cutaneous leishmaniasis

Diagnostic test	Positive	True negative	False negative	Sensitivity (%)	Specificity (%)	NPV (%)	PPV (%)
SSS	30	57	63	32.2	100	47.5	100
SE-RPA	61	57	32	65.5	100	64.04	100
ATL-PCR	86	57	7	92.4	100	89.06	100
RNAlater PCR	59	57	34	63.4	100	62.64	100

The total number of positive samples was 93 as determined by PCR. If any of these were found to be negative in a particular assay, it was considered as a false negative. NPV and PPV are negative and positive predictive values, respectively

## Discussion

Point of need diagnostics is an emerging need for the control of leishmaniases; it can help in active case detection in the field, surveillance and diagnosis of CL in low resource settings. Slit skin smear sensitivity is known to be low (35–70%) [4, 6, 13] with PCR being the most sensitive method in diagnosing CL and other types of leishmaniases. However, the disadvantages of the PCR method are that a punch biopsy PCR requires three hours of incubation for DNA extraction, a run time of 90 minutes or more depending upon the selected protocol, a need for high speed centrifugation, several steps of pipetting and another 45–60 minutes for electrophoresis and gel visualization. All these steps are quite cumbersome to perform and time consuming. In the case of real-time PCR, the post amplification procedure is not needed, but a complex expensive device is a must. The advantage of the rapid reverse purification method (SpeedXtract) and isothermal amplification technique (RPA) is that the whole DNA extraction and RPA method takes a maximum of 35 minutes, is easy to handle and results are available in real-time.

In the present study, punch biopsy was expected to produce the highest sensitivity with RPA, since it provides highest amount of sample (and hence the highest amount of DNA) compared to skin scrapings and FNA. Therefore, it was justifiable to use a punch biopsy (2 mm in diameter) as the ideal sample for RPA. The disadvantage of using a punch biopsy is that it has to be taken under local anaesthesia, in a hospital/clinic setting by a trained medical officer. This is a limitation in a field-based active case detection method. Therefore, a minimally invasive sample collection method would be needed in the future to make this mobile laboratory a complete field-based point of need diagnostic method for cutaneous leishmaniasis [14].

There is no doubt that the molecular assays (PCR, LAMP and RPA) detect *L. donovani* with higher diagnostic sensitivity than microscopy and culture [10, 12, 13, 15]. As shown here, the same PCR was strongly affected by the method of sample transportation. ATL-preserved samples outperformed the routine RNA*later* protocol. Since it is designed for RNA preservation, RNA*later* may not be the best transport medium for DNA preservation. Moreover, the lower sensitivity of RPA would still be due to the implementation of rapid DNA extraction method (SpeedXtract). As partially digested skin sample was observed at the end of recommended ten minutes compared to traditional three-hour spin column digestion using a DNeasy Blood and tissue kit. This may be due to the presence of keratin and fibrous tissue in skin. This argument supported by a study performed before, where both real-time PCR and RPA produced the same sensitivity using DNA extracted by SpeedXtract from blood samples of VL patients [10]. Furthermore, it was observed in our study that nine out of 25 SpeedXtract/RPA negative samples became RPA positive when RPA was performed with spin column extracted DNA (data not shown).

The use of the SpeedXtract-RPA assay at point of need has many advantages. Comparing the cost, where PCR cost 30 USD, a SpeedXtract-RPA only cost 10 USD per-reaction. When SpeedXtract/RPA is compared to another point of need diagnostic, e.g. antigen detection test (CL-Detect) [6], the SpeedXtract-RPA gave much higher sensitivity levels (65.5%) than CL-Detect (28%). Another advantage of SpeedXtract/RPA is that it did not give any false positive readings, giving it 100% specificity. Taking into account that the parasite load in the Sri Lankan CL lesions are low (median 2+) [6], a very sensitive method is needed to avoid false negatives. Therefore, SpeedXtract-RPA would be a good tool to diagnose low parasite density CL lesions as well as macular type PKDL lesions and to monitor the treatment response of CL and PKDL in the future.

An interesting observation made in this study is that there were 57 clinically suspected CL-lesions, which were negative in PCR, RPA and microscopy. These patients could not be followed up due to financial constraints and the different rural locations of these patients. These 57 patients could have differential diagnoses or could still be CL. This latter point would need further investigation in the future.

## Conclusions

In conclusion, molecular assays are strongly affected by the method of sample transportation and DNA extraction. RNA*later* should be avoided in any future sample transportation. SpeedXtract represents a promising technology for the rapid extraction of nucleic acid; nevertheless, further improvement of the protocol is needed to achieve better sensitivity. Furthermore, the introduction of an easy method for sample collection would make this SpeedXtract/RPA-based mobile suitcase method a complete field-based point of need diagnostic tool.

## Additional files

Additional file 1: **Figure S1.** Results of screening skin scrapping and aspirate with SpeedXtract (SE) and recombinase polymerase amplification (RPA) assay and punch biopsies with SE and RPA as well as DNeasy blood and tissue kit (Qiagen) and polymerase chain reaction (PCR). (PDF 1280 kb)

Additional file 2: **Table S1.** Results of screening of skin biopsies from 150 suspected CL cases with SpeedXtract/RPA, RNA*later*/PCR and ATL buffer/PCR, in addition to Giemsa-stained slit skin smears. (XLS 53 kb)

## Abbreviations

CL: Cutaneous leishmaniasis
FNA: Fine needle aspirate
LD: *Leishmania donovani*
PCR: Polymerase chain reaction
RPA: Recombinase polymerase amplification
SE: SpeedXtract
SSS: Slit skin smear
VL: Visceral leishmaniasis
