# Supplementary material for: Evaluation of rapid extraction and isothermal amplification techniques for the detection of Leishmania donovani DNA from skin lesions of suspected cases at the point of need in Sri Lanka
Source: Parasit Vectors. 2018 Dec 22;11:665. doi: 10.1186/s13071-018-3238-1 (PMC6303884; doi:10.1186/s13071-018-3238-1)
Supplement: Supplementary file 1 — Figure S1. Results of screening skin scrapping and aspirate with SpeedXtract (SE) and recombinase polymerase amplification (RPA) assay and punch biopsies with SE and RPA as well as DNeasy blood and tissue kit (Qiagen) and polymerase chain reaction (PCR). (PDF 1280 kb) [file 13071_2018_3238_MOESM1_ESM.pdf]

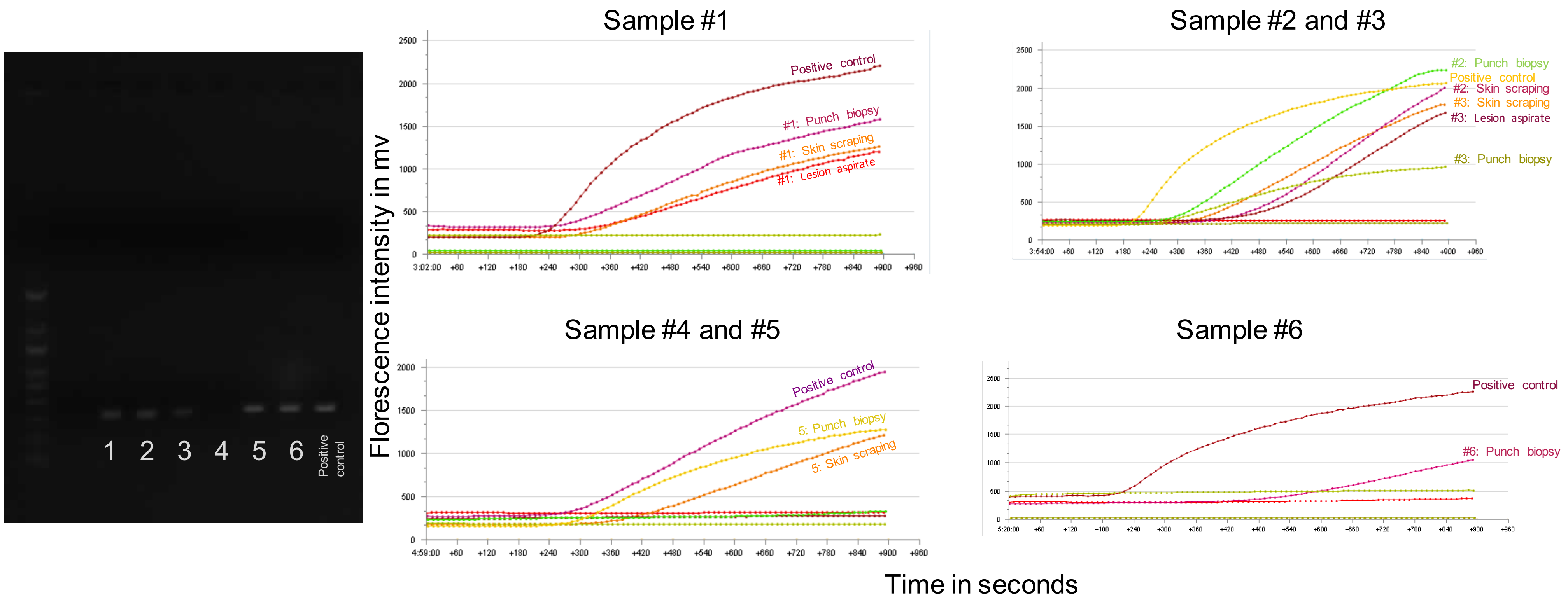

Additional file 1: Figure S1. Results of screening skin scrapping and aspirate with SpeedXtract (SE) and recombinase polymerase amplification (RPA) assay and punch biopsies with SE and RPA as well as DNeasy blood and tissue kit (Qiagen) and polymerase chain reaction (PCR).
